# The impact of risk perception on perceived benefits of generative artificial intelligence users—an empirical study based on university students

**DOI:** 10.3389/fpsyg.2026.1866428

**Published:** 2026-09-11

**Authors:** Wei Du, Shuhan Ning, Yukun Shi, Yongan Chen

**Affiliations:** 1 School of Economics and Management, North University of China, Taiyuan, Shanxi, China; 2 School of Education, Tianjin University, Tianjin, China; 3 School of Semiconductor and Physics, North University of China, Taiyuan, Shanxi, China

**Keywords:** generative artificial intelligence, perceived benefit, risk perception, threshold effect, university students

## Abstract

Amid the rapid integration of generative artificial intelligence (Gen AI) into higher education, it remains unclear how the risk perception ability of university students, as a core group of users, is associated with their perceived benefits of Gen AI. To address this gap, this study develops an integrated theoretical framework drawing upon Cognitive Appraisal Theory (CAT), Protection Motivation Theory (PMT), and the Technology Acceptance Model (TAM)/UTAUT2 to explain how different dimensions of risk perception differentially influence distinct categories of perceived benefits. Questionnaire surveys were conducted to collect valid data from 814 university students. Hierarchical regression analysis using SPSS was employed to examine the effects of demographic variables (gender, age, grade), Gen AI usage duration, and risk perception on perceived benefits. The results indicate that university students’ risk perception of Gen AI is multidimensional, and different dimensions show significantly differential associations with perceived benefits. Specifically, security risk perception has a significant positive effect on academic assistance and skill development. In contrast, information risk perception exerts significant negative effects on psychological and emotional support, daily life, and leisure and entertainment benefits. Results of the threshold effect show that when college students’ risk perception exceeds a certain threshold, the effect turns positive and significant, enhancing their perceived benefit. Based on these findings, it is recommended that related groups take actions to promote the development and application of Gen AI.

## 1 Introduction

Generative Artificial Intelligence (Gen AI), with advanced natural language processing and content generation capabilities, has revolutionized user–information system interaction and provided new avenues for information acquisition and processing. It has been integrated into university students’ daily learning, representing a transformative educational advancement. As early technology adopters, university students possess strong curiosity and adaptability; investigating their perceptions thus offers intuitive insights into technology adaptability. Recent studies on AI acceptance and readiness have provided robust empirical evidence for understanding how students form perceptions of AI technologies. Hamkah et al. (2025), drawing upon the Technology Acceptance Model (TAM), investigated factors shaping AI acceptance among engineering students in Indonesia and found that perceived usefulness, perceived ease of use, social influence, and self-efficacy significantly predicted AI acceptance, while perceived risks exerted a significant negative influence. Similarly, Sutrisno et al. (2025) examined Indonesian university students’ readiness for AI-based project learning that reinforces local wisdom values, revealing that students’ technological literacy, attitudes toward AI in learning, and awareness of cultural value integration collectively shape their preparedness to engage with AI-supported instructional environments. Extending this line of inquiry, Alkhwaldi et al. (2025b) employed a TAM-based approach to examine medical students’ intentions to use virtual reality for dynamic learning, further demonstrating the model’s applicability across diverse educational technologies and student populations. These studies underscore that AI readiness and acceptance are shaped by a complex interplay of cognitive, social, and cultural factors—a perspective that informs our investigation of how risk perception is associated with perceived benefits among Chinese university students. Risk-benefit dynamics in AI adoption have been observed across financial, agricultural, and academic contexts (Abdulmuhsin et al., 2026; Alkhwaldi et al., 2026; Tiwari et al., 2026), suggesting that the relationship between risk perception and perceived outcomes is not confined to student populations.

However, this integration, while enhancing text generation and information processing, has also introduced new challenges. Existing research indicates that student users face multidimensional risks when using Gen AI, including information risks from insufficient content accuracy, security risks from data privacy breaches, technical risks from system instability, and ethical and legal concerns such as academic integrity and intellectual property issues (García-López et al., 2025). Risk perception generally refers to individuals’ identification of objective dangers through intuitive judgment and subjective feeling (Zhang et al., 2024). Perceived benefit in our paper is distinct from related constructs in the technology acceptance literature. It differs from perceived usefulness (TAM), which narrowly focuses on task performance enhancement, and from perceived value (UTAUT2), which entails a cost-benefit calculus including monetary and psychological costs. Rather, perceived benefit refers to students’ holistic, multidimensional assessment of positive outcomes from Gen AI use across academic, emotional, skill-development, daily-life, and leisure domains. Unlike AI acceptance or adoption intention—behavioral predispositions toward technology use—perceived benefit is an evaluative judgment that can exist independently of actual usage decisions. This distinction shifts the research question from “whether students adopt Gen AI” to “what students gain from it”—a question that has received far less empirical attention. Evidently, university students’ risk perception of Gen AI potentially relates to both their adoption decisions and post-usage perceived benefits. University students’ perceived benefits typically involve assessing actual benefits from Gen AI interactions, including long-term development in behavioral improvement, skill enhancement, and emotional wellbeing (Han et al., 2025). The relationship between risk perception and perceived benefits is often complex and counterintuitive. Strong risk perception may lead students to avoid Gen AI, missing potential benefits; similarly, clear risk awareness may prompt more cautious and efficient use for academic growth and skill improvement. Moreover, this dynamic relationship evolves with user experience: students with longer exposure tend to identify potential risks more clearly while also more effectively harnessing benefits. Based on the above, this study employed a questionnaire survey, hierarchical regression analysis, and threshold regression analysis to examine how different dimensions of Gen AI risk perception are associated with university students’ perceived benefits. The contributions of this study are threefold. First, theoretically, this study extends existing Gen AI acceptance research by shifting attention from whether users adopt AI technologies to how users evaluate the benefits obtained from AI use under different risk perceptions. By integrating Cognitive Appraisal Theory, Protection Motivation Theory, and technology acceptance perspectives, this study develops a risk–benefit perspective to explain why different types of perceived risks may have differentiated associations with perceived benefits. The findings further reveal that risk perception is not uniformly detrimental; instead, its relationship with perceived benefits varies across risk dimensions and exhibits nonlinear patterns under certain conditions. Second, methodologically, this study applies threshold regression analysis to identify nonlinear relationships between Gen AI risk perception and perceived benefits, providing empirical evidence that the effects of perceived risks may change when risk perceptions reach different levels. Third, practically, this study provides differentiated implications for universities and related stakeholders by suggesting that AI risk management should not only focus on reducing perceived risks but also on improving students’ ability to understand, manage, and respond to AI-related risks. These findings contribute to a more nuanced understanding of university students’ experiences with Gen AI and provide insights for promoting effective and responsible AI use in higher education.

### 1.1 Literature review

#### 1.1.1 Risk perception of Gen AI among university students

Risk perception, originating in psychology, has been widely applied in technology acceptance, consumer behavior, e-commerce, and cybersecurity to explain concerns about technology adoption (Bauer, 1967; Slovic, 1987; Ogbanufe and Kim, 2018). With AI proliferation, it has become a key theoretical framework for understanding user attitudes toward emerging technologies.

University students’ risk perception of Gen AI can be categorized into five dimensions: information quality risk, security and privacy risk, ethical risk, technical limitations, and legal risk. Regarding information quality, Cotton et al. (2024) noted that distorted training data may generate false information, hindering students’ ability to verify knowledge and increasing screening burden, which further affects use attitudes (Kim and Lee, 2025). Students also worry about unauthorized data storage or sharing of personal information, undermining trust and use attitudes (Bukar et al., 2024; Stewart and Segars, 2002); Gen AI-generated content is also susceptible to plagiarism. Gen AI may generate biased or inappropriate content, raising academic ethical concerns (Zhang C. et al., 2025; Vistorte et al., 2024). Perceived technical risks include system instability (Venkatesh et al., 2016) and users’ insufficient ability to discern AI-generated content, which may exacerbate such risks (Khan et al., 2025). Legal risks center on intellectual property and personal information protection (Pellegrina and Helmy, 2025), with heightened concerns among graduate students due to stricter research norms.

#### 1.1.2 Perceived benefits of Gen AI among university students

Research indicates that university students experience positive effects from using Gen AI across multiple dimensions, including academic development, digital literacy, and psychological and emotional support and so on.

In academic development, Gen AI analyzes students’ learning styles and needs to provide customized content and feedback, improving learning experiences and task efficiency (Yang et al., 2025; Hao and Chen, 2025). It also streamlines information retrieval and data analysis, thereby enhancing research capabilities (Meakin, 2024). Regarding digital literacy, Gen AI fosters autonomy, creativity, critical and deep thinking—core skills for career development—and strengthens students’ ability to integrate complex data and evaluate information credibility (Zhang Z. et al., 2025; Meakin, 2024; Qi et al., 2025).

Gen AI also offers psychological and emotional support. Through personalized content and real-time feedback, it boosts student confidence and helps them cope with academic pressure (Lu and Lin, 2025). Students increasingly rely on such support during decision-making dilemmas. AI psychological counseling is positively perceived as a supplement to traditional services, particularly when resources are limited (Sun and Zhou, 2024; Hao et al., 2025).

Furthermore, Gen AI enhances innovation and interdisciplinary applications. It stimulates creative potential through content generation and personalized recommendations (Hao et al., 2025), and demonstrates unique value in fields such as architecture and biology (Geitmann and Bidhendi, 2023). Students develop multi-perspective thinking and active learning via AI-assisted writing and art (Tsao and Nogues, 2024; Conway and Smith, 2026). Gen AI also aids task planning, time management, and even fitness, improving exercise motivation and life management (Wang et al., 2025; Rawassizadeh et al., 2019).

Beyond these domain-specific benefits, emerging evidence suggests that AI can fundamentally enhance the quality of educational assessment and learning evaluation. Budiman et al. (2025) demonstrated that AI-driven assessments, through predictive technology and real-time feedback mechanisms, significantly improve assessment quality, enable personalized learning pathways, and foster skill development in vocational secondary school contexts. These findings contextualize the perceived academic benefits observed in our study—students may perceive Gen AI not merely as a convenience tool but as a personalized learning scaffold that enhances assessment quality and skill development.

The risk-benefit dynamics observed in education resonate across professional domains. Alkhwaldi et al. (2026) found that data security concerns inhibited AI adoption among farmers, echoing the deterrent effect of risk in our student sample. Abdulmuhsin et al. (2026) documented divergent effects of information versus security risk on academics’ LLM adoption—paralleling our dimension-specific hypotheses. Tiwari et al. (2026) showed that investor AI behavior involves a multidimensional risk-benefit calculus. This cross-population convergence—across students, academics, farmers, and investors—suggests that distinct risk dimensions trigger fundamental cognitive mechanisms in AI appraisal, lending external validity to our theoretical framework.

In summary, a review of the literature reveals that most existing studies treat risks and benefits as parallel independent variables, jointly examining their impact on adoption behavior. Furthermore, in most users’ psychological process of technology evaluation, there exists an underlying cognitive sequence: risk perception often serves as an initial, cautionary assessment. When users perceive potential threats posed by a technology, they naturally reassess the actual benefits it offers. Moreover, according to classical technology acceptance theory, risk is frequently viewed as an obstacle that benefits must overcome. Although current research has yielded considerable insights into college students’ risk perceptions and perceived benefits regarding Gen AI use, no closely related study has yet examined whether college students’ risk perceptions of Gen AI are associated with their perceived benefits. Therefore, this study treats perceived benefits as the dependent variable and risk perception, duration of Gen AI use, and demographic factors as the independent variables, employing multiple regression analysis to reveal potential associations.

### 1.2 Theoretical framework

Although the literature has separately examined risk perception and perceived benefits in the context of Gen AI, few studies have articulated a coherent theoretical mechanism explaining why and how different dimensions of risk perception exert differential effects on distinct categories of perceived benefits. To address this gap, this study integrates three complementary theoretical perspectives—Cognitive Appraisal Theory (CAT), Protection Motivation Theory (PMT), and the Technology Acceptance Model (TAM)/UTAUT2—to construct a unified theoretical framework that guides hypothesis development.

Cognitive Appraisal Theory (CAT), as the core integrative framework, originally developed by Lazarus and Folkman (1984), posits that individuals’ emotional and behavioral responses to a given situation are determined not by the objective features of the situation itself, but by their subjective cognitive appraisal of it. This appraisal process consists of two sequential stages: primary appraisal and secondary appraisal. In the context of this study, different dimensions of risk perception (information risk, security risk, technical risk, ethical risk, and legal risk) are conceptualized as distinct primary appraisals of threat. Students appraise specific types of risks differently depending on the nature of the threat and their prior experience. The critical theoretical innovation of applying CAT here lies in the secondary appraisal stage: when students perceive a risk as controllable—that is, when they believe they have the knowledge, skills, or resources to mitigate the risk—they are more likely to engage in active, problem-focused coping. Conversely, when they perceive a risk as uncontrollable—when they feel powerless to prevent negative outcomes. Thus, CAT think that risk perception does not directly translate into perceived benefits; rather, it operates through the mediating pathway of secondary appraisal (perceived controllability) and subsequent coping strategies.

Protection Motivation Theory (PMT) complements CAT by specifying the cognitive processes underlying threat appraisal and coping appraisal. In our study, PMT helps explain why certain risk perceptions negatively affect perceived benefits, while others positively affect them. When threat appraisal is high but coping appraisal is low (e.g., a student perceives high information risk but lacks the skills to verify AI-generated content), the result is maladaptive avoidance or anxiety, which undermines perceived benefits in domains like emotional support or daily life. However, it’s the opposite.

Technology Acceptance Model (TAM) identifies perceived usefulness as a key determinant of technology adoption, defined as the degree to which a user believes that using a technology would enhance their job performance. Our construct of perceived benefit is broader and more comprehensive than perceived usefulness. UTAUT2 extends TAM by introducing additional predictors such as hedonic motivation (the fun or enjoyment derived from using a technology) and price value (the perceived trade-off between benefits and costs). These constructs are particularly relevant for explaining perceived benefits in non-academic domains. The applicability of the UTAUT framework has been validated beyond educational settings; for instance, Alkhwaldi et al. (2025a) employed the UTAUT model to investigate consumer acceptance of AI-driven fintech services, demonstrating its utility in understanding technology adoption across diverse sectors. Meanwhile, perceived benefit differs from AI acceptance or adoption intention, which are behavioral predispositions toward using a technology. Perceived benefit is an evaluative judgment that can exist independently of actual usage decisions—a student may perceive high benefits from Gen AI but still choose not to use it due to other constraints, or may use it regularly while perceiving only modest benefits.

Therefore, integrating these three theoretical perspectives, we propose the following causal logic: 1. Risk perception dimensions (information, security, technical, ethical, legal) serve as primary appraisals of threat (CAT). 2. Students then engage in secondary appraisal (CAT), evaluating whether the threat is controllable and whether they have adequate coping resources. 3. PMT specifies the dual pathways: if coping appraisal is high, threat perception triggers adaptive, problem-focused coping (e.g., learning, skill development, cautious usage); if coping appraisal is low, it triggers maladaptive, emotion-focused coping (e.g., anxiety, avoidance). 4. Adaptive coping is expected to be associated with higher perceived benefits; maladaptive coping is expected to be associated with lower perceived benefits. 5. The relationship between risk perception and perceived benefit is therefore non-linear and dimension-specific: the same level of risk may produce positive or negative effects depending on the perceived controllability of that specific risk type and the student’s coping self-efficacy.

The expectation of threshold effects for security and technical risks is grounded in PMT and the Yerkes-Dodson law (Yerkes and Dodson, 1908). PMT suggests that coping appraisal activates only when threat perception reaches a sufficient level; below this threshold, individuals remain complacent. The Yerkes-Dodson law posits that moderate arousal optimizes cognitive engagement. Thus, security and technical risks may exhibit threshold patterns rather than linear associations with perceived benefits.

This integrated framework provides the theoretical foundation for the hypotheses developed in the following section, moving beyond empirical observation to theory-driven prediction.

### 1.3 Hypothesis development

Drawing upon the integrated theoretical framework, we derive the following hypotheses from the logical principles of CAT, PMT, and TAM/UTAUT2.

#### 1.3.1 Security risk and academic skill benefits

Security risk concerns unauthorized data collection, privacy breaches, and personal information misuse. According to CAT, this risk type is controllabl—students can manage it through measures such as using anonymous accounts, avoiding sensitive information disclosure, and reviewing privacy policies. When secondary appraisal indicates controllability, problem-focused coping is activated—learning data protection practices and developing prudent usage habits. PMT further posits that, given high coping efficacy, threat perception translates into adaptive protection motivation, accompanying more deliberate usage and skilled practitioners, thereby showing positive associations with academic assistance.

*H1a*: Security risk perception is positively associated with perceived academic assistance benefits.

*H1b*: Security risk perception is positively associated with perceived skill development benefits.

#### 1.3.2 Information risk and emotional/daily-life benefits

Information risk refers to concerns about the accuracy and reliability of AI-generated content. This risk type has low controllability, as verifying AI output requires substantial external resources. CAT predicts that low controllability triggers emotion-focused coping; PMT indicates that high threat combined with low coping efficacy elicits anxiety and avoidance. This distrust undermines domains requiring emotional investment (psychological support, daily decision-making) and hedonic motivation (UTAUT2)-driven leisure experiences, thus showing negative associations with related benefits.

*H2a*: Information risk perception is negatively associated with perceived psychological and emotional support benefits.

*H2b*: Information risk perception is negatively associated with perceived daily life benefits.

*H2c*: Information risk perception is negatively associated with perceived leisure and entertainment benefits.

#### 1.3.3 Technical risk and threshold effect

Security risk and technical risk involve concerns about data privacy, system opacity, and instability. Both are moderately controllable through learning and practice. Drawing on PMT, we posit that low risk perception fails to trigger coping, yielding limited benefits; moderate-to-high risk perception activates coping appraisal, prompting deliberate usage and skill acquisition. The Yerkes-Dodson law provides a complementary mechanism: moderate arousal optimizes cognitive engagement. Hence, the associations are nonlinear: below critical thresholds, they are weak or negative; above them, positive.

*H3*: Security risk and technical risk perception exhibit threshold effects on perceived benefits (academic assistance, skill development, and psychological and emotional support), such that the associations become positive only after risks exceed critical levels.

#### 1.3.4 Other risk dimensions

Ethical risk and legal risk, concerns about biased content and copyright liability, vary in controllability across individuals. Given their exploratory nature in this context, we do not propose directional hypotheses for these dimensions but will examine their associations empirically.

## 2 Materials and methods

### 2.1 Variable measurement

To ensure the transparency and rigor of our measurement approach, we followed established scale development guidelines. The initial item pool for each construct was generated through a deductive approach, drawing upon extensively validated instruments from prior studies. All items were adapted to reflect the specific context of Gen AI use among university students. For risk perception, items were adapted from Song et al. (2021), Dinev and Hart (2006), Boillat et al. (2022), Schepman and Rodway (2020), and Gerlich (2023); for perceived benefits, items were adapted from Baidoo-Anu and Ansah (2023), Gao et al. (2018), Choi and Drumwright (2021), Vakkari et al. (2016), and Sun et al. (2023). To establish content validity, the preliminary item pool was reviewed by three subject-matter experts in educational technology and measurement, who evaluated each item for relevance, clarity, and representativeness of the target construct. Based on their feedback, several items were reworded for clarity, and redundant items were eliminated. A pilot study was then conducted with 45 university students (not included in the final sample) to assess face validity, item comprehensibility, and completion time. Based on pilot feedback, minor wording adjustments were made to improve clarity. The final instruments demonstrated satisfactory psychometric properties as reported in the Results section.

#### 2.1.1 Independent variable measurement

The independent variable comprises five dimensions of Gen AI risk perception. This study drew on research methods of Kirova et al. (2023), Wach et al. (2023), Al-kfairy et al. (2024), and Lyu (2023), incorporated risk perception theory (Lan, 2023), and considered university students’ purposes, contexts of Gen AI use, and group characteristics. On this basis, risk perception was divided into five core dimensions: information risk, security risk, technical risk, ethical risk, and legal risk. These dimensions reflect Gen AI’s inherent characteristics and encompass its potential impacts at individual and legal levels. To ensure reliability and validity, each risk dimension was assessed through multiple measurement items adapted from previously validated scales.

Specifically, the information risk dimension reflects users’ concerns about the accuracy and authenticity of Gen AI-generated content, distinguishing Gen AI from traditional AI systems and stemming from information quality and reliability issues arising from its autonomous content generation capabilities (Lyu, 2024). The security risk dimension embodies concerns about privacy breaches and illegal data sharing. Gen AI’s model of processing large amounts of personal data to generate personalized content intensifies data security and privacy protection risks. The technical risk dimension focuses on perceptions of technical characteristics, including over-reliance and diminished judgment due to system inexplicability, as well as behavioral unpredictability from autonomous learning. Additionally, ethical risk involves concerns about generated content violating moral values, blurred human-machine boundaries, and lack of emotional empathy (Yang, 2024). The legal risk dimension addresses legal consequences of content infringement and sharing, encompassing legal challenges such as copyright attribution, liability determination, and content regulation (Yang, 2024). Specific measurement items are presented in Table 1 (Zhao et al., 2025). Each item was assessed using a 7-point Likert scale (1 = strongly disagree, 7 = strongly agree).

**TABLE 1:** Risk perception dimensions and measurement items for Gen AI among university student users.

Risk dimension	Item ID	Measurement item	Reference source
Information risk	IR1	I am concerned that the sources of content generated by Gen AI may be inaccurate or contain errors.	(Song et al., 2021)
	IR2	I am concerned that content generated by Gen AI may be repetitive and homogeneous.	
	IR3	I am concerned that content generated by Gen AI may contain false, misleading, or erroneous information.	
	IR4	I am concerned that Gen AI may generate content that appears reliable but cannot withstand scrutiny or lacks logical consistency.	
Security risk	SR1	I am concerned that my personal information and data may be collected, leaked, or misused without authorization when using Gen AI.	(Dinev and Hart, 2006)
	SR2	I am concerned that Gen AI may share or leak the content I generate without my consent.	
	SR3	I am concerned that the encryption and storage methods used by Gen AI for personal information or data are not secure enough and may be vulnerable to hacking.	
Technical risk	TR1	I find it difficult to understand how Gen AI generates content, which makes me doubt its reliability.	(Boillat et al., 2022)
	TR2	I believe Gen AI lacks clear feedback or correction request mechanisms, which reduces my trust in it.	
	TR3	I am concerned that Gen AI may encounter unknown errors during generation (e.g., unstable network), leading to inaccurate or incomplete content.	
	TR4	I am concerned that over-reliance on Gen AI may weaken my ability to judge potential system errors and risks.	
Ethical risk	ER1	I am concerned that Gen AI might (intentionally or unintentionally) generate misleading or deceptive content.	(Schepman and Rodway, 2020)
	ER2	I am concerned that Gen AI might generate content that violates moral values (e.g., gender discrimination, etc.).	
	ER3	I am concerned that Gen AI might blur the boundaries between human-generated and machine-generated content (e.g., misleading users or causing copyright disputes).	
	ER4	I am concerned that Gen AI might generate ethical and moral decisions that should only be made by humans.	
Legal risk	LR1	I am concerned that there might be legal consequences if content generated by Gen AI infringes intellectual property.	(Gerlich, 2023)
	LR2	I am concerned that users sharing or using Gen AI content containing false information might face legal liability.	
	LR3	I am concerned that users sharing or using Gen AI content containing misleading information might face legal liability.	
	LR4	I am concerned that there are insufficient legal frameworks to regulate and hold developers and users of Gen AI accountable for their actions.	

#### 2.1.2 Dependent variable measurement

The dependent variable is university students’ perceived benefits of Gen AI (Han et al., 2025). Based on the literature review in section 2.2, combined with application scenarios of Gen AI products such as Wenxin Yiyan (a large language model independently developed in China) and ChatGPT and preliminary understanding of user experiences, this study divided perceived benefits into five dimensions: academic assistance, skill development, psychological and emotional support, daily life, and leisure and entertainment. Each dimension was treated as a separate variable, with items designed to understand whether students benefit from Gen AI services across different dimensions. Specific measurement items are presented in Table 2. Each item was measured using a 5-point Likert scale (1 = never, 2 = unclear, 3 = rarely, 4 = sometimes, 5 = often). It should be noted that the reasons for using the 7-point and 5-point Likert scales in this article are as follows. Risk perception involves nuanced cognitive evaluations of potential threats across multiple dimensions, requiring finer-grained discrimination among response options. Empirical evidence suggests that 7-point scales provide greater variability and reduced response skewness compared to 5-point scales. Given that risk perception served as the primary independent variable in our hierarchical regression analyses, we prioritized measurement precision for this construct. Conversely, perceived benefits were measured as frequency of experienced outcomes using a 5-point scale with clearly labeled anchors. For frequency-based behavioral assessments, 5-point scales provide sufficient discrimination while offering easier response processing. Moreover, prior validation studies for the benefit measures we adapted employed 5-point formats, and we maintained consistency with the original instruments to preserve their established psychometric properties. Importantly, both scales demonstrated excellent internal consistency (Cronbach’s α> 0.93), and the clear factor structures obtained via exploratory factor analysis suggest that the different scale formats did not compromise measurement quality or introduce systematic bias.

**TABLE 2:** Dimensions and measurement items of perceived benefit.

Perceived benefit dimension	Item ID	Measurement item	Reference source
Academic assistance	AA1	I believe Gen AI can help me improve the quality of my academic writing (e.g., essays, research papers).	(Baidoo-Anu and Ansah, 2023)
	AA2	I believe Gen AI can help me understand complex academic concepts and content.	
	AA3	I believe Gen AI can help me prepare for exams and quizzes more effectively.	
	AA4	I believe Gen AI can assist me with my research tasks.	
	AA5	I believe Gen AI can help stimulate my creativity and generate ideas for research or projects.	
Skill development	SD1	I believe Gen AI can help me develop critical thinking by providing diverse perspectives and analytical approaches.	(Gao et al., 2018)
	SD2	I believe Gen AI can help me identify and improve weaknesses in my learning process.	
	SD3	I believe Gen AI helps me enhance specialized skills required in my professional field.	
	SD4	I believe Gen AI can help me develop better problem-solving strategies in my research area.	
	SD5	I believe Gen AI can help me improve my communication and presentation skills.	
Psychological and emotional support	PES1	I believe Gen AI can provide supportive interactions to reduce loneliness during independent study.	(Choi and Drumwright, 2021)
	PES2	I believe Gen AI can provide emotional support when I feel academic pressure.	
	PES3	I believe Gen AI can help me develop strategies to cope with academic stress.	
	PES4	I believe Gen AI can help boost my confidence in facing academic challenges.	
Daily life	DL1	I believe Gen AI can help me better organize my daily schedule.	(Vakkari et al., 2016)
	DL2	I believe Gen AI can help me make wiser decisions in daily activities.	
	DL3	I believe Gen AI can provide me with health-related lifestyle suggestions.	
	DL4	I believe Gen AI can enhance my campus life experience.	
	DL5	I believe Gen AI can provide me with effective time or money management advice.	
Leisure and entertainment	LE1	I believe Gen AI can recommend entertainment content suited to my preferences (e.g., books, movies, games).	(Sun et al., 2023)
	LE2	I believe Gen AI can help me cultivate new interests and hobbies.	
	LE3	I believe Gen AI’s suggestions can help me better participate in campus activities.	
	LE4	I believe Gen AI can help me find campus activities that match my interests.	

This 5-point frequency scale was adopted to maintain consistency with the original validated instruments and to facilitate participant response processing for behavioral frequency assessments.

### 2.2 Data collection

**FIGURE 1:**
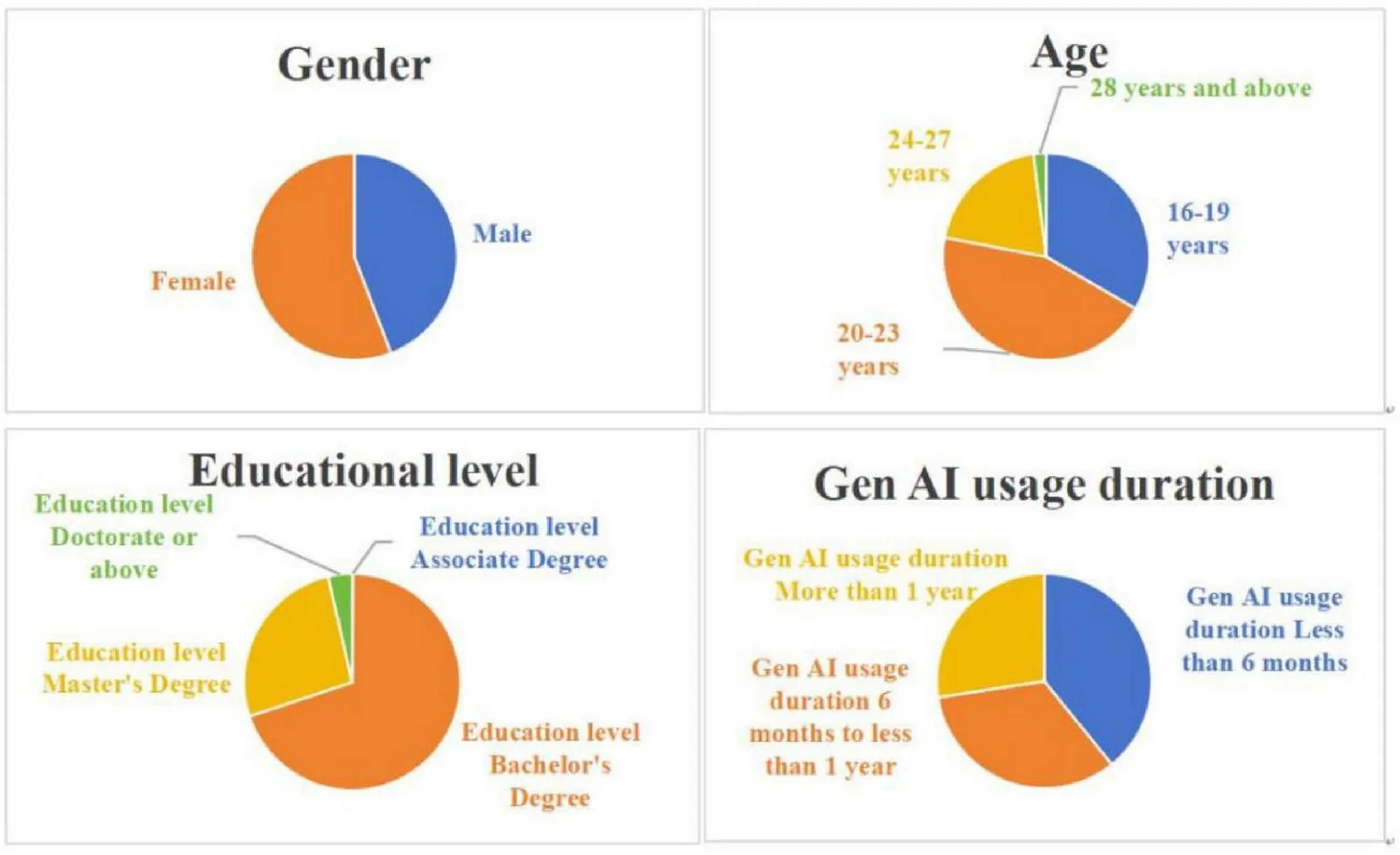
Sample characteristics chart.

Questionnaires were distributed from late October to mid-November 2024, yielding 2613 responses via Wen Juan Xing. After excluding respondents who had never used Gen AI (*n* = 1,071) and invalid responses (excessively short completion times or logical contradictions, *n* = 728), 814 valid questionnaires were obtained, yielding a valid response rate of 31.2%. The sample covered users of different ages, educational levels, and familiarity with Gen AI technology, ensuring broad representativeness. Demographic characteristics of sample are presented in Figure 1.

### 2.3 Data analysis methods

**FIGURE 2:**
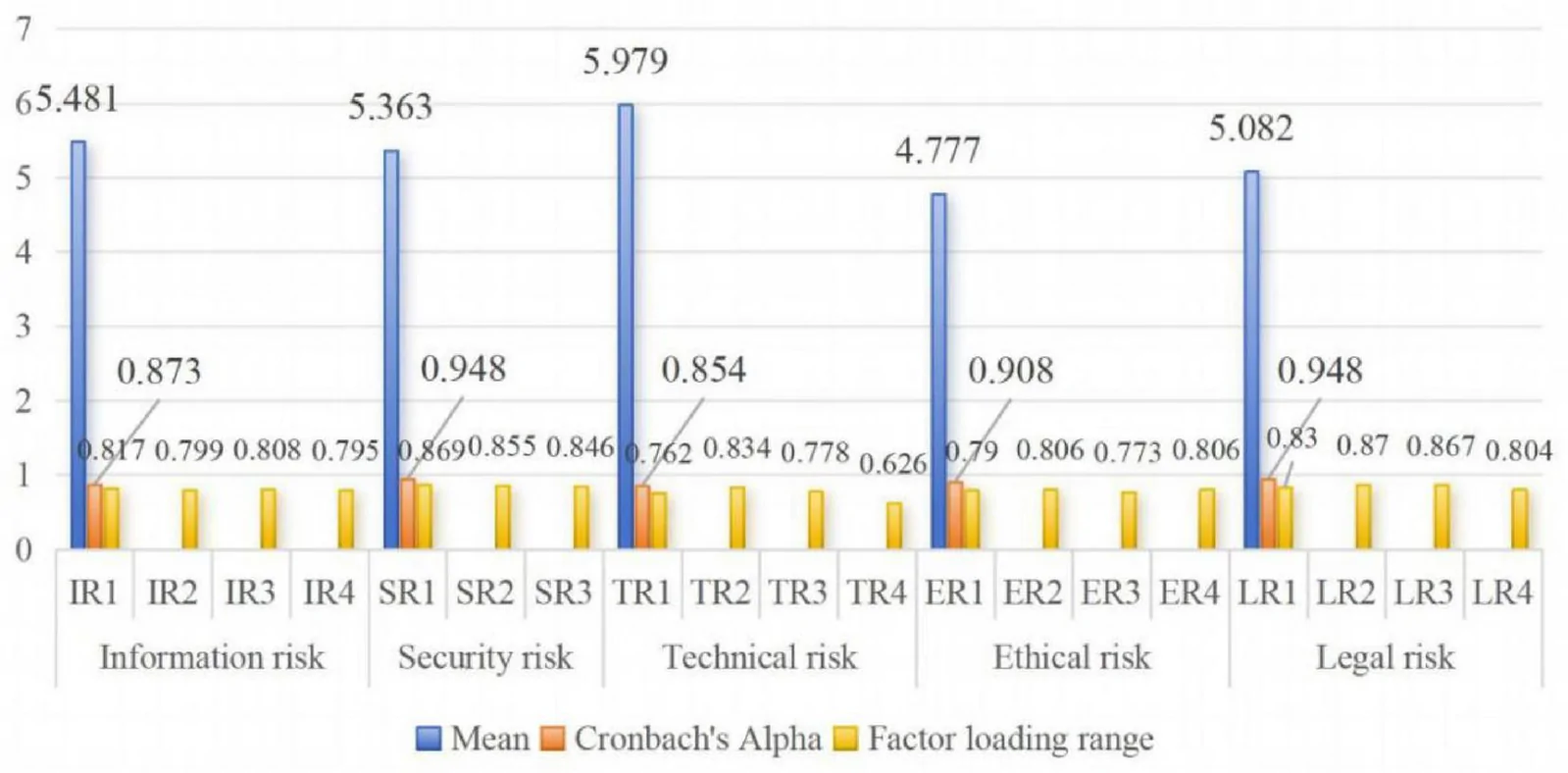
Graph of the reliability and validity test results for independent variables.

**FIGURE 3:**
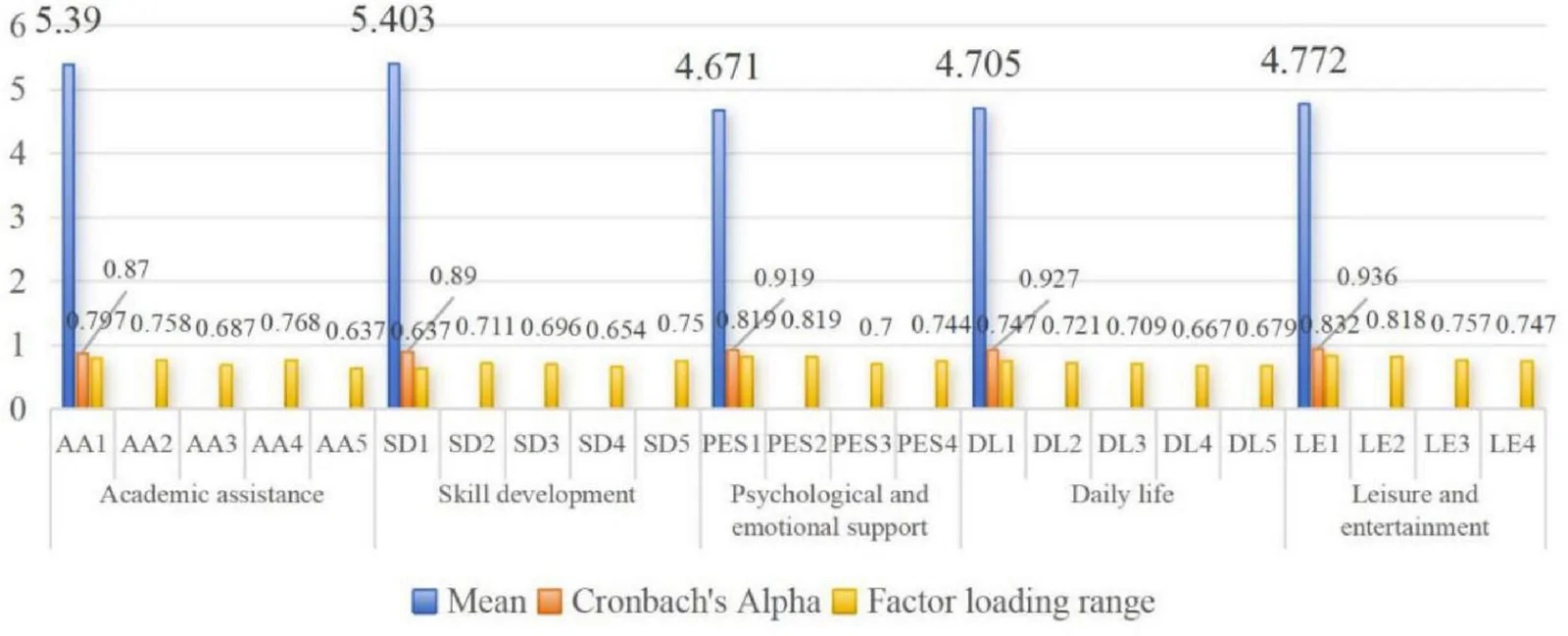
Graph of the reliability and validity test results for dependent variables.

SPSS 23.0 was employed for data analysis. First, Cronbach’s α coefficients were calculated to test internal consistency (α > 0.7 indicates good reliability). Concurrently, exploratory factor analysis (EFA) was conducted on risk perception and perceived benefit dimensions to examine validity and ensure adequate discrimination. Principal component analysis was employed for factor extraction, followed by varimax orthogonal rotation, retaining items with factor loadings > 0.5. Second, hierarchical regression analysis within multiple linear regression was employed. This study established three-layer regression models: Layer 1 examined effects of control variables (gender, age, education level) on perceived benefit dimensions; Layer 2 added Gen AI usage duration; Layer 3 added the core risk perception variables. It is important to clarify why hierarchical regression was employed rather than structural equation modeling (SEM), given that the core constructs are theoretically multidimensional and the sample size (*N* = 814) is sufficient for SEM. This analytical choice reflects a deliberate paradigm distinction rather than a methodological oversight. First, the research question determines the analytical paradigm. This study is fundamentally prediction-oriented rather than theory-testing in the latent variable sense. The primary objective is to quantify the incremental predictive power of distinct risk perception dimensions over specific perceived benefit outcomes—that is, to assess how much additional variance each risk block explains after controlling for covariates. Hierarchical regression is purpose-built for this stepwise variance partitioning and provides direct interpretability of ΔR^2^ and standardized coefficients. SEM, by contrast, is optimally designed for testing global model fit and structural path networks, which is not the central focus here. Second, the methodological innovation of this study—threshold regression—is exclusively regression-based. The core contribution lies in identifying nonlinear activation effects via Hansen’s (2000) threshold estimation, which requires piecewise regression with an indicator function. This procedure is well-validated in conventional regression frameworks but remains statistically underdeveloped in SEM contexts. Adopting hierarchical regression ensures methodological coherence across the two analytical stages (variance partitioning and threshold detection), whereas mixing SEM with threshold regression would introduce technical inconsistency. Third, the measurement approach provides sufficient empirical support for using composite scores as reliable proxies. All scales were adapted from extensively validated instruments (see Tables 1, 2). In the present sample, EFA yielded clear factor structures with all loadings > 0.6, cross-loadings below 0.4/0.5, cumulative variance explained > 75%, and Cronbach’s α > 0.93 for both scales. To further justify the use of mean composite scores in our regression analyses, we examined the item-level psychometric properties across all dimensions. As shown in Figures 2, 3, Cronbach’s α values ranged from 0.736 to 0.869 for risk dimensions and from 0.570 to 0.819 for benefit dimensions, with all factor loadings exceeding 0.56 and corrected item-total correlations above 0.60. These indicators, combined with the EFA results, support the reliability of composite scoring for prediction-oriented regression and threshold estimation (Shmueli, 2010; Hair et al., 2019). While confirmatory factor analysis (CFA) would provide additional evidence regarding the dimensional structure and construct validity of the proposed measures, the present study adopts composite scores as observed indicators because the primary objective is to examine the predictive relationships and threshold effects among risk perception dimensions and perceived benefits. The use of composite scores is consistent with a prediction-oriented analytical approach, where theoretically derived constructs are operationalized through aggregated measures rather than latent variable modeling. In this study, the satisfactory factor loadings, reliability coefficients, and exploratory factor analysis (EFA) results provide empirical support for the appropriateness of using composite scores in subsequent regression and threshold analyses. Similar approaches have been adopted in educational psychology and technology acceptance research, where validated scales are aggregated to examine predictive relationships among theoretically meaningful constructs (e.g., Fu et al., 2025; Hamkah et al., 2025). Nevertheless, we acknowledge that CFA-based validation could further strengthen the assessment of convergent and discriminant validity, and future research should employ CFA or structural equation modeling (SEM) to further validate and extend the present findings. Nevertheless, using observed composite scores does ignore measurement error, and the absence of standalone CFA means that convergent and discriminant validity (e.g., CR, AVE, HTMT) were not formally tested. These are genuine methodological limitations. The study explicitly acknowledges them in the Limitations section, where future CFA-based SEM is recommended to cross-validate the regression-based findings. This analytical choice thus reflects a deliberate trade-off: prioritizing prediction-oriented hypothesis testing and methodological coherence with the threshold model over full latent-variable modeling, while fully recognizing the cost of this decision.

## 3 Results

### 3.1 Reliability and validity analysis

The overall reliability of the risk perception scale was 0.937, indicating good reliability. The KMO value was 0.917, and Bartlett’s test was significant (χ^2^ = 6258.882, df = 171, *p* < 0.001), indicating suitability for factor analysis. Similarly, as described above, EFA extracted five factors comprising 19 items, with each factor containing three or more items. Total variance explained reached 79.582%. The rotated component matrix showed all items had factor loadings > 0.6 on their respective factors and < 0.4 on other factors, indicating good convergent and discriminant validity. Results are presented in Figure 2.

The reliability of the perceived benefit scale was 0.955, indicating high reliability. The KMO value was 0.949, and Bartlett’s test was significant (χ^2^ = 7623.786, df = 253, *p* < 0.001). Similarly, EFA extracted five factors comprising 23 items, with each factor containing three or more items. Total variance explained reached 75.991%. The rotated component matrix showed all items had factor loadings > 0.6 on their respective factors and < 0.5 on other factors, indicating good convergent and discriminant validity. Results are presented in Figure 3.

### 3.2 Multiple regression analysis

This study employed hierarchical regression analysis to examine effects of demographic variables, Gen AI usage duration, and risk perception on different dimensions of perceived benefits. Results are presented in Tables 3, 4. Variance inflation factors (VIF) for all models were below 5, indicating no multicollinearity issues.

**TABLE 3:** Regression analysis results for academic assistance and skill development.

Statistical variables	Academic assistance dimension			Skill development dimension		
	Model 1	Model 2	Model 3	Model 4	Model 5	Model 6
	*B*	*B*	*B*	*B*	*B*	*B*
(Constant)	6.107***	5.703***	5.221***	5.765***	5.420***	4.922***
Gender (1 = Male)	0.201	0.168	0.206	0.074	0.043	0.077
Age	−0.043	−0.035	−0.038	−0.022	−0.015	−0.017
Education level						
Master’s degree	0.335*	0.279	0.283	0.179	0.129	0.130
Doctoral degree or above	0.376	0.301	0.246	0.075	0.012	−0.053
Gen AI usage duration						
0.5–1 year		0.238	0.225		0.183	0.172
> 1 year		0.617***	0.612***		0.544***	0.539***
Risk perception factors			−0.066			−0.064
Information risk			0.134*			0.131*
Security risk			0.047			0.062
Technical risk			−0.108			−0.100
Ethical risk			0.089			0.066
Legal risk						
*F*	2.090	5.478***	4.010***	0.531	3.431**	2.781**

*p < 0.10, **p < 0.05, ***p < 0.01

**TABLE 4:** Regression analysis results of psychological and emotional support, daily-life and leisure and entertainment.

Statistical variables	Psychological and emotional support dimension			Daily life dimension			Leisure and entertainment dimension		
	Model 7	Model 8	Model 9	Model 10	Model 11	Model 12	Model 13	Model 14	Model 15
	*B*	*B*	*B*	*B*	*B*	*B*	*B*	*B*	*B*
Constant	4.434***	4.307***	4.319***	4.888***	4.779***	4.759***	4.702***	4.627***	4.653***
Gender (1 = Male)	0.117	0.086	0.118	0.173	0.154	0.155	0.126	0.126	0.134
Age	0.012	0.016	0.015	−0.011	−0.009	−0.007	0.005	0.007	0.005
Education level									
Master’s degree	−0.419	−0.451*	−0.438*	−0.315	−0.337	−0.336	−0.541**	−0.547**	−0.544**
Doctoral degree or above	−0.343	−0.364	−0.400	0.078	0.059	−0.028	−0.431	−0.445	−0.508
Gen AI usage duration									
0.5–1 year		−0.090	−0.062		−0.019	−0.003		0.093	0.102
>1 year		0.339	0.450*		0.240	0.368*		0.072	0.174
Informat-ion risk			−0.288**			−0.258**			−0.217*
Security risk			0.134			0.106			0.131
Technical risk			0.113			0.053			−0.018
Ethical risk			−0.042			0.119			0.095
Legal risk			0.086			−0.010			0.018
Model fit indicators									
*F*	1.569	2.091	2.068*	1.842	1.705	1.873*	3.274*	2.231*	1.921*

*p < 0.10, **p < 0.05, ***p < 0.01.

#### 3.2.1 Academic assistance as dependent variable

After estimating Models 1, 2, and 3 using the sample data, the results revealed that: in Model 1, education level exerted a significant effect: education level exerted a significant effect: the association between Gen AI use and academic assistance was significantly stronger for master’s students than for doctoral students (β = 0.335, *p* < 0.10). The pattern that may reflect differences in academic experience and research demands rather than a causal effect of educational level *per se*. Results from Models 2 and 3 indicated that Gen AI usage duration had a significant effect: respondents with > 1 year of use perceived significantly higher academic assistance benefits than those with < 6 months (β = 0.612, SE = 0.130, *p* < 0.05). Gender, age, and education level did not exert significant effects. Regarding risk perception, security risk perception had a significant positive association (β = 0.134, SE = 0.061, *p* < 0.05).

#### 3.2.2 Skill development as dependent variable

Regression analyses of Models 4, 5, and 6 using the sample data showed that: Gen AI usage duration had a significant effect: respondents with > 1 year of use perceived significantly higher skill development benefits than those with < 6 months (β = 0.539, SE = 0.132, *p* < 0.05). Gender, age, and education level did not exert significant effects. Security risk perception had a significant positive association (β = 0.131, SE = 0.062, *p* < 0.05).

In summary, results are presented in Table 3. The effect of Gen AI on academic assistance was significantly stronger for master’s students than for doctoral students. Respondents with > 1 year of usage perceived significantly higher academic assistance and skill development benefits than those with < 6 months. Security risk perception showed significantly positive associations with both perceived academic assistance and skill development benefits.

#### 3.2.3 Psychological and emotional support as dependent variable

When the sample data were regressed on Models 7, 8, and 9, the results indicated that education level exerted a significant effect: master’s students perceived significantly higher psychological and emotional support benefits than doctoral students (β = −0.438, SE = 0.218, *p* < 0.05). This observed difference may be associated with variations in academic experience and research expectations across educational levels, rather than indicating a direct causal influence of educational status. Gen AI usage duration had a significant effect: respondents with > 1 year of use perceived significantly higher benefits than those with < 6 months (β = 0.450, SE = 0.178, *p* < 0.05). Gender and age did not exert significant effects. Information risk perception had a significant negative association (β = −0.288, SE = 0.101, *p* < 0.05).

#### 3.2.4 Daily life as dependent variable

After estimating Models 10, 11, and 12 using the sample data, the results showed that Gen AI usage duration had a significant effect: respondents with > 1 year of use perceived significantly higher daily life benefits than those with < 6 months (β = 0.368, SE = 0.170, *p* < 0.05). Gender, age, and education level did not exert significant effects. Information risk perception had a significant negative association (β = −0.258, SE = 0.096, *p* < 0.05).

#### 3.2.5 Leisure and entertainment as dependent variable

Regression analyses of Models 13, 14, and 15 using the sample data argued that Education level exerted a significant effect: master’s students perceived significantly higher leisure and entertainment benefits than doctoral students (β = −0.544, SE = 0.210, *p* < 0.05). This pattern may be attributable to differences in usage contexts or individual preferences rather than a systematic effect of educational level. Gender, age, and Gen AI usage duration did not exert significant effects. Information risk perception had a significant negative association (β = −0.217, SE = 0.097, *p* < 0.05).

In summary, results are presented in Table 4. Education level showed significant associations with perceived psychological and emotional support benefits and perceived leisure and entertainment benefits. Gen AI usage duration had significant effects on psychological and emotional support benefits and daily life benefits. Information risk perception demonstrated significant negative correlations with perceived psychological and emotional support, daily life, and leisure and entertainment benefits.

### 3.3 Common method bias

Given that all variables were collected via self-report questionnaires from the same respondents at a single time point, Harman’s single-factor test was conducted to assess common method bias. All measurement items were entered into an unrotated principal component analysis. As shown in Table 5, the first factor accounted for 36.687% of the total variance, well below the recommended threshold of 40% (Podsakoff et al., 2003). And the second factor contributing an additional 33.025% (cumulative 69.712%). This two-factor structure aligns with the theoretical distinction between risk perception and perceived benefits as two primary constructs, further supporting that common method variance is not a serious concern.

**TABLE 5:** Total variance explained in Harman’s single-factor test.

Component	Initial eigenvalues		
	Total	% of Variance	Cumulative %
Information risk	3.669	36.687	36.687
Security risk	3.302	33.025	69.712
Technical risk	0.822	8.215	77.927
Ethical risk	0.570	5.700	83.627
Legal risk	0.372	3.720	87.347
Academic assistance	0.338	3.377	90.724
Skill development	0.309	3.085	93.809
Psychological and emotional support	0.248	2.481	96.291
Daily life	0.194	1.944	98.235
Leisure and entertainment	0.177	1.765	100.000

### 3.4 Further analysis

To further investigate under what conditions and to what extent college students’ risk perception is associated with their perception of benefits, this study draws on the research by Mei and Xie (2021) to construct a threshold effect model based on survey data, with the aim of addressing the aforementioned questions and deepening the analysis.

#### 3.4.1 Threshold model specification

Drawing on the research methods of Zhao et al. (2020) and Li et al. (2022), this paper constructs a threshold effect model, which is as follows:


$${\text{Y}}_{\text{i}}=\alpha_{0}+\alpha_{1}{\text{X}}_{\text{i}}\times I\left(\mathrm{Th}\leq \mathrm{q1}\right)+\alpha_{2}{\text{X}}_{\text{i}}\times I\left(\mathrm{Th}>\mathrm{q1}\right)$$ (1)

$$\;\;+\alpha_{3}\,\mathrm{Controls}+\varepsilon_{\text{i}}$$

In Equation (1), Y_*i*_ represents perceived benefits; X_*i*_ represents risk perception and the threshold variable; I(⋅) is an indicator function taking the values 1 or 0, where it is 1 if the condition in parentheses is satisfied, and 0 otherwise; Controls denotes the control variables in this article, namely gender, age, educational attainment, and Gen AI usage duration; ε_*i*_ is the random disturbance term; i indexes the individual. Equation (1) considers a single-threshold scenario and can be extended to a multi-threshold scenario based on steps such as econometric tests of the sample data. Candidate threshold values were theoretically anchored in PMT and the Yerkes-Dodson law. The midpoint of the 7-point Likert scale (4) and its adjacent values were pre-specified as theoretically plausible candidates, reflecting the transition from low to moderate risk awareness. Final thresholds were empirically estimated via Hansen’s (2000) bootstrap method, with the theoretical range as a guiding constraint.

#### 3.4.2 Analysis of threshold regression results

Since the data used are cross-sectional in this paper, and in order to test for and analyze threshold effects, this study follows the approach of Hansen (2000) by first using the Bootstrap method to test the number and values of the thresholds. After 5,000 bootstrap samples, the corresponding *p*-values were obtained. The specific test results are shown in Table 6. Since risk perception serves as a general term for the independent variables in the study, the independent variables have been renamed for the sake of statistical analysis and observation: information risk is denoted as X1, security risk as X2, technical risk as X3, ethical risk as X4, and legal risk as X5. Similarly, the dependent variables have been renamed as follows: academic assistance is denoted by Y1, skill development by Y2, psychological and emotional support by Y3, daily life by Y4, and leisure and entertainment by Y5.

**TABLE 6:** Table testing the threshold effect of risk perception on perceived benefit.

Variables	Academic assistance (Y1)			Skill development (Y2)			Psychological and emotional support (Y3)		
	Threshold type	Threshold value	*P*-value	Threshold type	Threshold value	*P*-value	Threshold type	Threshold value	*P*-value
Security risk (X2)	Single threshold	3	0.0004	Single threshold	3	0			
	Double threshold	4	0.0186						
Technical risk (X3)	Single threshold	4.75	0	Single threshold	4.75	0	Single threshold	2.75	0.0002

Obtain the *p*-value by conducting 5,000 repeated samplings using the bootstrap method; *p* < 0.1 indicates the presence of a threshold effect.

Results of Table 6 show that there is a double-threshold effect between X1 and X2, with threshold values of 3 and 4, separately. X2 has a single-threshold effect on Y2, with a threshold value of 3. X3 exhibits a single-threshold effect on Y1, Y2, and Y3, with threshold values of 4.75, 4.75, and 2.75, respectively. Other variables not included in Table 5 were excluded because they did not exhibit a threshold effect during the threshold effect test.

The results in Table 6 indicate the presence of a threshold effect. To further examine how students’ perceptions of risk and the benefits of Gen AI differ before and after this threshold, we conducted a threshold regression analysis on the relevant data, with results presented in Tables 7, 8.

**TABLE 7:** Threshold regression results on the impact of security risk with academic assistance and skill development.

Variables	Academic assistance (Y1)			Skill development (Y2)	
	X2⋅I(Th ≤ 3)	X2⋅I(3<Th<4)	X2⋅I(Th ≥ 4)	X2⋅I(Th ≤ 3)	X2⋅I(Th>3)
Security risk (X2)	0.477	1.245	0.304***	0.346	0.166***
	(0.321)	(0.646)	(0.069)	(0.312)	(0.055)
95% confidence interval	[−0.270, 1.106]	[−1.017, 2.511]	[0.014, 0.591]	[−0.530, 0.957]	[0.057, 0.493]
Controls	YES	YES	YES	YES	YES
*N*	36	121	657	36	778
*F*	16.415***	15.884***	82.046***	9.976***	74.084***

The standard error is shown in parentheses. Threshold estimates were obtained via Hansen’s (2000) bootstrap method with 5,000 replications. Controls include gender, age, education level, and Gen AI usage duration. ***p < 0.01. X2 = Security risk.

**TABLE 8:** Threshold regression findings on the influence of technical risk on academic assistance, skill development, and psychological and emotional support.

Variables	Academic assistance (Y1)		Skill development (Y2)		Psychological and emotional support (Y3)	
	X3⋅I(Th ≤ 4.75)	X3⋅I(Th>4.75)	X3⋅I(Th ≤ 4.75)	X3⋅I(Th>4.75)	X3⋅I(Th ≤ 2.75)	X3⋅I(Th>2.75)
Technical risk (X3)	−0.325***	0.432***	−0.356	0.414***	1.966	0.211**
	(0.101)	(0.105)	(0.095)	(0.113)	(0.603)	(0.082)
95% confidence interval	[−0.522, −0.019]	[0.136,0.863]	[−0.828,0.041]	[0.132,0.636]	[−0.570,3.148]	[0.047,0.641]
Controls	YES	YES	YES	YES	YES	YES
*N*	337	477	337	477	27	787
*F*	38.818***	73.277***	41.917***	62.221***	37.557***	37.807***

The standard error is shown in parentheses. Threshold estimates were obtained via Hansen’s (2000) bootstrap method with 5,000 replications. Controls include gender, age, education level, and Gen AI usage duration. **p < 0.05, ***p < 0.01. X3 = Technical risk.

Based on the threshold regression results presented in Table 7 for the impact of security risk on academic assistance and skill development, first, with respect to academic support: when safety risks are at their lowest level, the estimated coefficient is 0.477, but this result is not statistically significant. When security risk fall within the intermediate range, the coefficient increases to 1.245, but the result remains statistically insignificant. When security risk exceed the second threshold and reach the highest range, the coefficient becomes 0.304 and is significant at the 1% level. These findings indicate that college students’ perception of security risk does not significantly influence academic help-seeking in the low- or medium-risk ranges. Only when safety risks accumulate to a relatively high level do they significantly promote academic assistance. Although the coefficients in the low- and medium-risk ranges are positive but not statistically significant, the significant positive result in the high-risk range suggests that the positive effect of security risk exhibits a threshold effect: only when risks are sufficiently high is it likely to trigger students’ academic assistance behavior. Second, the regression results concerning college students’ perception of security risk and skill development show that when security risk are below the threshold, the coefficient is 0.346 and is not statistically significant. When it exceeds the threshold, the regression coefficient becomes 0.166 and is statistically significant at the 1% level (*p* < 0.01). These findings suggest that the positive effect of security risk on skill development emerges only when risk levels are relatively high. Compared to academic assistance, the impact of security risk on skill development is relatively minor, indicating that once the initial barrier to entry is crossed, security risks promote direct help-seeking behavior more strongly than they foster skill development.

The threshold regression results presented in Table 8 examine the associations of technical risk with academic assistance, skill development, and psychological and emotional support. The results are as follows: First, when technical risk is below the threshold value of 4.75, the coefficient for its impact on academic assistance is -0.325 and is significant at the 1% level. This suggests that in the low-risk phase, college students’ perception of technical risk has a significant inhibitory effect on academic assistance. Once technical risk exceeds the threshold value and enters the high-risk range, the coefficient changes to 0.432, remaining significant at the 1% level. This shows that in the high-risk stage, technical risk exerts a significant positive promoting effect on academic assistance. These findings imply that the impact of technical risk on academic assistance follows a U-shaped pattern. When risk levels are low, students may reduce their academic assistance behavior due to technical barriers or concerns about usage. However, once the risk accumulates to a certain extent, it may actually trigger coping-oriented assistance behavior, thereby positively promoting academic assistance. Second, when technical risk is below the threshold value of 4.75, the coefficient of its impact on skill development is -0.356, but its result is not statistically significant. When technical risk exceeds the threshold value, the coefficient becomes 0.414 and is statistically significant at the 1% level. These results suggest that the promotional effect of college students’ perception of technical risk on skill development only becomes evident when risk levels are relatively high. A possible explanation is that moderate technical risk challenges may prompt college students to actively explore and master relevant skills, aiming to enhance their own abilities through the process of addressing these risks. Third, when technical risk falls below the threshold value of 2.75, its effect on psychological and emotional support is not significant. However, once technical risk enters the high-risk range, the coefficient becomes 0.211 and is statistically significant. Thus, at higher levels of technological risk, a significant positive effect on psychological and emotional support emerges. In summary, when risk levels are low, the effects of security risk and technical risk on academic assistance, skill development, and psychological and emotional support are mostly negative or insignificant. However, once risk levels exceed the threshold and enter the high-risk range, these effects all shift to being significantly positive. This suggests that risk perception has a dual nature in educational contexts: moderate or even high levels of risk may be accompanied by coping behaviors, which in turn are associated with proactive assistance. It also indicates that, under specific conditions, risk can serve as a trigger for students’ active adaptation and development.

#### 3.4.3 Robustness and sensitivity analysis

To enhance the persuasiveness of the threshold findings, we conducted robustness tests by altering the trimming proportion and sensitivity tests by excluding control variables. Tables 9, 10 present the corresponding results.

**TABLE 9A:** Results of robustness tests for threshold regression (Trimming = 0.1).

Variables	Academic assistance (Y1)		
	X2⋅I(Th ≤ 3)	X2⋅I(3<Th<4)	X2⋅I(Th ≥ 4)
(A) Security risk			
Security risk (X2)	0.477	1.245	0.304***
	(0.321)	(0.646)	(0.069)
95% confidence interval	[−0.270, 1.106]	[−1.017, 2.511]	[0.014, 0.591]
Controls	YES	YES	YES
*N*	36	121	657
*F*	2.902***	3.077***	16.256***

The standard error is shown in parentheses. Threshold estimates were obtained via Hansen’s (2000) bootstrap method with 5,000 replications. Controls include gender, age, education level, and Gen AI usage duration. **p < 0.05, ***p < 0.01. X2 = Security risk.

**TABLE 9B:** 

Variables	Academic assistance (Y1)		Skill development (Y2)	
	X3⋅I(Th ≤ 4.75)	X3⋅I(Th ≥ 4.75)	X3⋅I(Th ≤ 6.5)	X3⋅I(Th>6.5)
(B) Technical risk				
Technical risk (X3)	−0.262	0.413***	0.070	5.277**
	(0.108)	(0.105)	(0.053)	(2.170)
95% confidence interval	[−0.473, 0.134]	[0.028, 6.722]	[−0.049, 0.174]	[1.023, 9.531]
Controls	YES	YES	YES	YES
*N*	304	474	718	60
*F*	6.329***	15.491***	10.554***	6.992***

The standard error is shown in parentheses. Threshold estimates were obtained via Hansen’s (2000) bootstrap method with 5,000 replications. Controls include gender, age, education level, and Gen AI usage duration. **p < 0.05, ***p < 0.01. X3 = Technical risk.

**TABLE 10:** Sensitivity analysis of threshold-based results.

Variables	Academic assistance (Y1)		Skill development (Y2)		Psychological and emotional support (Y3)	
	X3⋅I(Th ≤ 4.5)	X3⋅I(Th>4.5)	X3⋅I(Th ≤ 6.5)	X3⋅I(Th>6.5)	X3⋅I(Th ≤ 6.5)	X3⋅I(Th>6.5)
Technical risk(X3)	−0.168	0.366	0.142***	4.814**	0.226**	6.183
	(0.161)	(0.102)	(0.055)	(1.898)	(0.089)	(4.333)
95% confidence interval	[−0.555, 0.259]	[−0.251, 5.224]	[0.034, 0.250]	[1.094, 8.533]	[0.052, 0.937]	[−2.310, 14.676]
Controls	NO	NO	NO	NO	NO	NO
*N*	83	371	394	60	394	60
*F*	2.120*	10.049***	3.317***	5.918***	2.318***	2.119

The standard error is shown in parentheses. Threshold estimates were obtained via Hansen’s (2000) bootstrap method with 5,000 replications. *p < 0.1, **p < 0.05, ***p < 0.01. X3 = Technical risk.

To assess the sensitivity of threshold estimates to sample trimming, we varied the trimming proportion from 0.15 to 0.1 following Hansen (2000) and got the outcomes of the Table 9. The results indicate that varying the trimming proportion does not alter the substantive conclusions. Consistent with the threshold regression findings, security risk and technical risk continue to exert significant positive effects on academic assistance once the thresholds are crossed. Technical risk also maintains a significant positive effect on skill development above the threshold. These cross-validation results reinforce the robustness of our conclusions.

We performed sensitivity analyses by excluding control variables (Table 10). The results show that technical risk continued to exert a positive effect on academic assistance after crossing the threshold. Its positive effects on skill development and psychological and emotional support also remained. These findings corroborate the robustness of our conclusions.

Regarding concerns about small subsamples (e.g., *n* = 27 for technical risk below 2.75 on psychological support in Table 6), three mitigating factors are noteworthy: (a) the Bootstrap procedure (5,000 replications) provides bias-corrected standard errors robust to small-sample distortions; (b) the threshold existence *p*-values (e.g., *p* = 0.0002 in Table 6) are far below conventional significance levels; and (c) results are consistent across different trimming and specification choices. These checks confirm that our threshold findings are not artifacts of specific modeling choices.

## 4 Discussion

### 4.1 Interpretation of findings

#### 4.1.1 Security risk and academic assistance: controllability activates problem-focused coping

Security risk is positively associated with academic assistance (β = 0.134, *p* < 0.05) and skill development (β = 0.131, *p* < 0.05). This pattern is explicable via Cognitive Appraisal Theory (CAT). Security risks are controllable—students can adopt protective measures (e.g., privacy policy review). Perceived controllability triggers problem-focused coping: learning data practices, developing cautious habits, and deepening Gen AI understanding. Protection Motivation Theory (PMT) further clarifies that high coping efficacy transforms threat into adaptive protection motivation, enhancing deliberate engagement. This aligns with Fu et al. (2025), who documented a “benefit-risk-coping paradox” whereby risk perception does not uniformly suppress engagement. Our finding extends this literature by demonstrating that security risk can facilitate benefit perception post-adoption—a mechanism distinct from its deterrent effect on initial adoption.

#### 4.1.2 Information risk and psychological and emotional support: low controllability triggers emotion-focused coping

The negative associations between information risk and psychological and emotional support (β = −0.288, *p* < 0.05), daily life (β = −0.258, *p* < 0.05), and leisure/entertainment benefits (β = −0.217, *p* < 0.05) are explained by the same framework. Unlike security risks, information risks—concerns about content accuracy and reliability—are largely uncontrollable; verification requires substantial external effort unavailable to most students. Low controllability triggers emotion-focused coping; PMT specifies that high threat with low coping efficacy produces maladaptive avoidance, eroding trust in domains requiring emotional investment.

This pattern is consistent with Melchior and Farinosi (2026), who surveyed 1,347 students across 24 Italian universities and found that cognitive and learning risks were the most frequently cited disadvantages of Gen AI, significantly undermining perceived personal and social benefits. Similarly, Buizza et al. (2026) reported that Italian university students’ concerns about AI-generated content reliability reduced willingness to engage with AI tools in non-academic contexts. These convergent findings suggest that the detrimental effect of information risk on emotional and hedonic outcomes is robust across cultural contexts. Budiman et al. (2025) found that information risk undermines trust in AI feedback, reducing psychological benefits. Our study extends this by showing that only information risk—not security or technical risk—negatively affects emotionally invested domains, underscoring the need for a multidimensional approach to risk perception. The study by Benabbes et al. (2026) on the use of AI and critical thinking in higher education in political science further supports our argument. The risk-benefit patterns observed in our student sample resonate across domains. Alkhwaldi et al. (2026) found that data security concerns inhibited farmers’ AI adoption, while policy support facilitated positive perceptions. Abdulmuhsin et al. (2026) documented divergent effects of information versus security risk on academics’ LLM adoption—paralleling our dimension-specific findings. Tiwari et al. (2026) showed that investor AI behavior involves a multidimensional risk-benefit calculus. This cross-domain convergence suggests that dimension-specific risk effects are not unique to education but reflect broader human-AI interaction patterns across professional and personal contexts.

#### 4.1.3 Threshold effects of security and technical risks: activation through coping appraisal

The threshold effects for security and technical risks can be interpreted through a dual-activation mechanism integrating Protection Motivation Theory (PMT) and the Yerkes-Dodson law.

First, at the cognitive-appraisal level (PMT), the threshold reflects the differential activation of threat appraisal versus coping appraisal. Threat appraisal can be activated at low risk levels, but coping appraisal requires a higher cognitive threshold. Below the threshold (e.g., technical risk < 4.75 for academic assistance), students acknowledge the risk but lack motivational impetus, resulting in avoidance (β = −0.325, *p* < 0.01). Above the threshold, perceived severity forces students to evaluate their coping resources, transforming threat awareness into benefit-seeking (β = 0.432, *p* < 0.01).

Second, at the neuropsychological level (Yerkes-Dodson law), the threshold corresponds to the optimal point of cognitive arousal. Low risk perception produces insufficient arousal, leading to superficial engagement. Moderate-to-high risk perception elevates arousal to an optimal zone, sharpening attention and promoting deliberate usage. Notably, the threshold for psychological and emotional support (2.75) is lower than that for academic assistance (4.75), suggesting that emotional domains require less arousal to trigger benefit perception.

Integrating these two lenses, we propose that risk facilitates benefit perception only when (1) threat is severe enough to activate coping appraisal and (2) arousal reaches an optimal level. Below this dual-activation threshold, risk generates anxiety without productive coping; above it, risk-driven arousal, channeled through coping appraisal, promotes adaptive engagement. This mechanism explains the threshold patterns observed in Tables 7, 8, offering a psychologically grounded account of why risk is not uniformly detrimental.

### 4.2 Limitations

These findings extend technology acceptance theories by revealing when risk facilitates rather than hinders benefit perception, and offer methodological and practical implications for evidence-based AI integration in higher education. Several limitations should be acknowledged. First, the cross-sectional design precludes causal inference; all reported relationships should be interpreted as statistical associations. The directionality of these relationships remains unclear without longitudinal or experimental data. Future research should employ time-lagged or experimental designs to test the causal pathways suggested by our theoretical framework.

Second, the sample was limited to Chinese university students, which may restrict generalizability to other populations or cultural contexts.

Third, the selection of control variables was relatively limited; we did not include potential confounders such as AI literacy, digital competence, frequency of use, academic discipline, or prior training. Future studies should incorporate these variables to isolate the unique role of risk perception.

Fourth, as noted in the section 2.3, the analytical strategy prioritized predictive hypothesis testing and threshold effect estimation over full latent-variable modeling. While our instruments demonstrated satisfactory reliability and factorial structure via EFA, and were adapted from well-validated sources, we treated construct scores as observed composite means rather than latent factors in the regression analyses. This approach ignores measurement error in the independent and dependent variables, which may lead to attenuated or biased coefficient estimates. We did not perform CFA or report CR, AVE, or HTMT, which limits the strength of construct-level validity claims. Future research should employ CFA-based SEM to simultaneously account for measurement error and structural relationships, thereby extending and cross-validating our regression-based findings.

Fifth, common method bias may exist due to the self-report, single-time-point data collection. Harman’s single-factor test was conducted, and the first factor accounted for 36.687% of the total variance—below the recommended 40% threshold. However, this diagnostic procedure has limited sensitivity in detecting common method variance, and more rigorous procedures—such as marker-variable analysis or common latent factor analysis—were not performed. The absence of these additional diagnostics means that the reported associations could be partially inflated or deflated by common method variance, a possibility that should be considered when interpreting the findings. Future research should incorporate procedural remedies (e.g., temporal separation of predictor and criterion measurement) and statistical remedies (e.g., marker-variable adjustment or common latent factor modeling) to more rigorously control for this bias.

### 4.3 Practical implications

To better integrate Gen AI with higher education, this study proposes the following recommendations.

(1) Universities should reframe security risk awareness as a pedagogical asset. Security risk perception is positively associated with academic assistance (β = 0.134, *p* < 0.05) and skill development (β = 0.131, *p* < 0.05), suggesting that concerned students engage more deliberately with Gen AI. Universities should integrate data privacy education into AI literacy curricula to transform passive worry into active, informed engagement.

(2) Developers should prioritize content transparency to counter information risk. Information risk perception shows significant negative associations with psychological and emotional support (β = −0.288, *p* < 0.05), daily life (β = −0.258, *p* < 0.05), and leisure and entertainment benefits (β = −0.217, *p* < 0.05). Developers should enhance source attribution, fact-checking indicators, and confidence scores to rebuild user trust in emotionally invested domains.

(3) A threshold-based strategy is needed for technical risks. Technical risk shifts from negative to positive association with academic assistance and skill development once it exceeds the critical value of 4.75 (β:−0.325 → 0.432, both *p* < 0.01); for psychological and emotional support, the threshold is 2.75. Rather than uniformly minimizing technical risks, universities and developers should provide structured training that helps students cross this “activation threshold”, turning technical challenges into learning opportunities. To this end, universities should design scaffolded AI literacy programs that safely guide students across the threshold without inducing disengagement. A “zone of productive risk” exists—wherein concerns are sufficient to activate coping appraisal (PMT) and optimal arousal (Yerkes–Dodson), yet not so high as to provoke avoidance. Instructional designers can operationalize this zone via three evidence-informed strategies: structured exposure (progressing from low-stakes retrieval to critical creation), cognitive reappraisal training (reframing technical limitations as learnable challenges, thereby shifting secondary appraisal per CAT and enhancing perceived controllability), and collaborative skill-building workshops (verification, debugging, and peer-assisted learning). Collectively, these scaffolding measures elevate risk perception past the threshold while equipping students with adequate coping resources, thus preventing the disengagement observed when high risk coincides with low coping capacity.

(4) Usage experience amplifies benefits. Students with over 1 year of Gen AI use consistently report higher benefits across multiple dimensions. Universities should offer early introductory courses, and policymakers should fund AI literacy initiatives to accelerate experiential learning.

(5) Policymakers should adopt differentiated frameworks. Given that security risk positively predicts academic skill outcomes while information risk negatively predicts emotional/daily-life benefits, one-size-fits-all regulations are inadequate. Policymakers should develop distinct standards for information quality, data protection, and ethical oversight, supported by collaborative governance platforms involving universities, developers, and government agencies.

In summary, universities, developers, and policy makers should work collaboratively to create favorable conditions for applying Gen AI in education, enabling students and other stakeholders to fully benefit from this emerging technology.

## 5 Conclusion

First, university students demonstrated high concern for information risk, security risk, and legal risk, but relatively low concern for technical risk and ethical risk. This can be explained at multiple levels. At the individual level, as frequent Internet users, students often encounter personal information breaches, fraud, and defamation. At the institutional level, universities have introduced cybersecurity measures that have raised student awareness. At the policy level, improved cyber regulations have enhanced students’ understanding of legal consequences. In contrast, technical and ethical risks are less directly experienced—students tend to prioritize efficiency-enhancing tools and, unless engaged in technology development, rarely attend to technical issues. Their lower concern for ethical issues may also stem from greater immediate worry about tangible risks like information leakage.

Second, hierarchical regression analysis revealed a significant effect of Gen AI usage duration. With the exception of leisure and entertainment benefits, students who had used Gen AI for more than 1 year perceived significantly higher benefits than those with less than 6 months of use. This suggests that greater technical competence and usage experience enable students to derive more value from Gen AI.

Third, the inclusion of risk perception did not substantially increase the explanatory power for perceived benefits, indicating that while risk perception plays a role, its influence is limited. Specifically, security risk perception had a significant positive association with academic assistance and skill development. When students perceive stronger security risks, they may develop greater learning motivation, handle data and research processes more cautiously, and acquire new skills to mitigate threats—thereby enhancing both research quality and personal competence. In contrast, information risk perception showed a significant negative association with psychological and emotional support, daily life, and leisure and entertainment benefits. Stronger information risk perception heightens privacy concerns, reduces willingness to disclose personal information, and leads students to worry about leakage, misuse, or identity theft. These concerns consequently coincide with their perceived benefits in non-academic domains.

The results of the threshold effect analysis indicate that at low risk levels, safety risks and technical risks mostly exert negative or insignificant effects on academic assistance, skill development, and psychological and emotional support. However, once risk levels cross the threshold and enter the high-risk range, these impacts all shift to significant positive effects. This suggests that, under specific conditions, risk may serve as a trigger mechanism for college students’ proactive adaptation and development.

Caveat on interpretation. The findings are based on cross-sectional data and should be interpreted as statistical associations, not causal relationships. While our theoretical framework suggests plausible causal pathways—whereby risk perception triggers coping appraisals that shape perceived benefits—confirming these directions requires longitudinal or experimental designs. The implications discussed above should be read with this methodological constraint in mind.

## Data Availability

The raw data supporting the conclusions of this article will be made available by the authors, without undue reservation.
